# Ready for malaria elimination: zero indigenous case reported in the People’s Republic of China

**DOI:** 10.1186/s12936-018-2444-9

**Published:** 2018-08-29

**Authors:** Jun Feng, Li Zhang, Fang Huang, Jian-Hai Yin, Hong Tu, Zhi-Gui Xia, Shui-Sen Zhou, Ning Xiao, Xiao-Nong Zhou

**Affiliations:** 1 National Institute of Parasitic Diseases, Chinese Center for Disease Control and Prevention, Shanghai, People’s Republic of China; 2 Key Laboratory of Parasite and Vector Biology, Ministry of Health, Shanghai, People’s Republic of China; 3 WHO Collaborating Centre for Tropical Diseases, Shanghai, People’s Republic of China; 4 National Center for International Research on Tropical Diseases, Shanghai, People’s Republic of China

**Keywords:** Malaria, Indigenous case, Zero, China

## Abstract

**Background:**

Malaria was once one of the most serious public health problems in China. However, the disease burden has sharply declined and epidemic areas have shrunk after the implementation of an integrated malaria control and elimination strategy, especially since 2000. In this review, the lessons were distilled from the Chinese national malaria elimination programme and further efforts to mitigate the challenges of malaria resurgence are being discussed.

**Methods:**

A retrospective evaluation was performed to assess the changes in malaria epidemic patterns from 1950 to 2017 at national level. The malaria data before 2004 were collected from paper-based annual reports. After 2004, each of the different cases from the Infectious Diseases Information Reporting Management System (IDIRMS) was closely examined and scrutinized. An additional documenting system, the National Information Management System for Malaria, established in 2012 to document the interventions of three parasitic diseases, was also examined to complete the missing data from IDIRMS.

**Results:**

From 1950 to 2017, the occurrence of indigenous malaria has been steeply reduced, and malaria-epidemic regions have substantially shrunk, especially after the launch of the national malaria elimination programme. There were approximately 30 million malaria cases annually before 1949 with a mortality rate of 1%. A total of 5999 indigenous cases were documented from 2010 to 2016, with a drastic reduction of 99% over the 6 years (2010, n = 4262; 2016, n = 3). There were indigenous cases reported in 303 counties from 18 provinces in 2010, but only 3 indigenous cases were reported in 2 provinces nationwide in 2016. While in 2017, for the first time, zero indigenous case was reported in China, and only 7 of imported cases were in individuals who died of *Plasmodium falciparum* infection.

**Conclusion:**

Malaria elimination in China is a country-led and country-owned endeavour. The country-own efforts were a clear national elimination strategy, supported by two systems, namely a case-based surveillance and response system and reference laboratory system. The country-led efforts were regional and inter-sectoral collaboration as well as sustained monitoring and evaluation. However, there are still some challenges, such as the maintenance of non-transmission status, the implementation of a qualified verification and assessment system, and the management of imported cases in border areas, through regional cooperation. The findings from this review can probably help improving malaria surveillance systems in China, but also in other elimination countries.

**Electronic supplementary material:**

The online version of this article (10.1186/s12936-018-2444-9) contains supplementary material, which is available to authorized users.

## Background

Today, the world still faces great challenges in fighting the scourge of malaria. According to the 2017 World Health Organization (WHO) World Malaria Report, after a period of unprecedented global success in malaria control, progress has stalled [1]. In 2016, 91 countries reported a total of 216 million cases of malaria, an increase of 5 million cases over the previous year, while the number of malaria deaths reached 445,000 [1]. Malaria transmission occurred mainly in areas where resources are limited, and local health systems are weak and cannot provide adequate diagnosis and treatment.

**Fig. 1:**
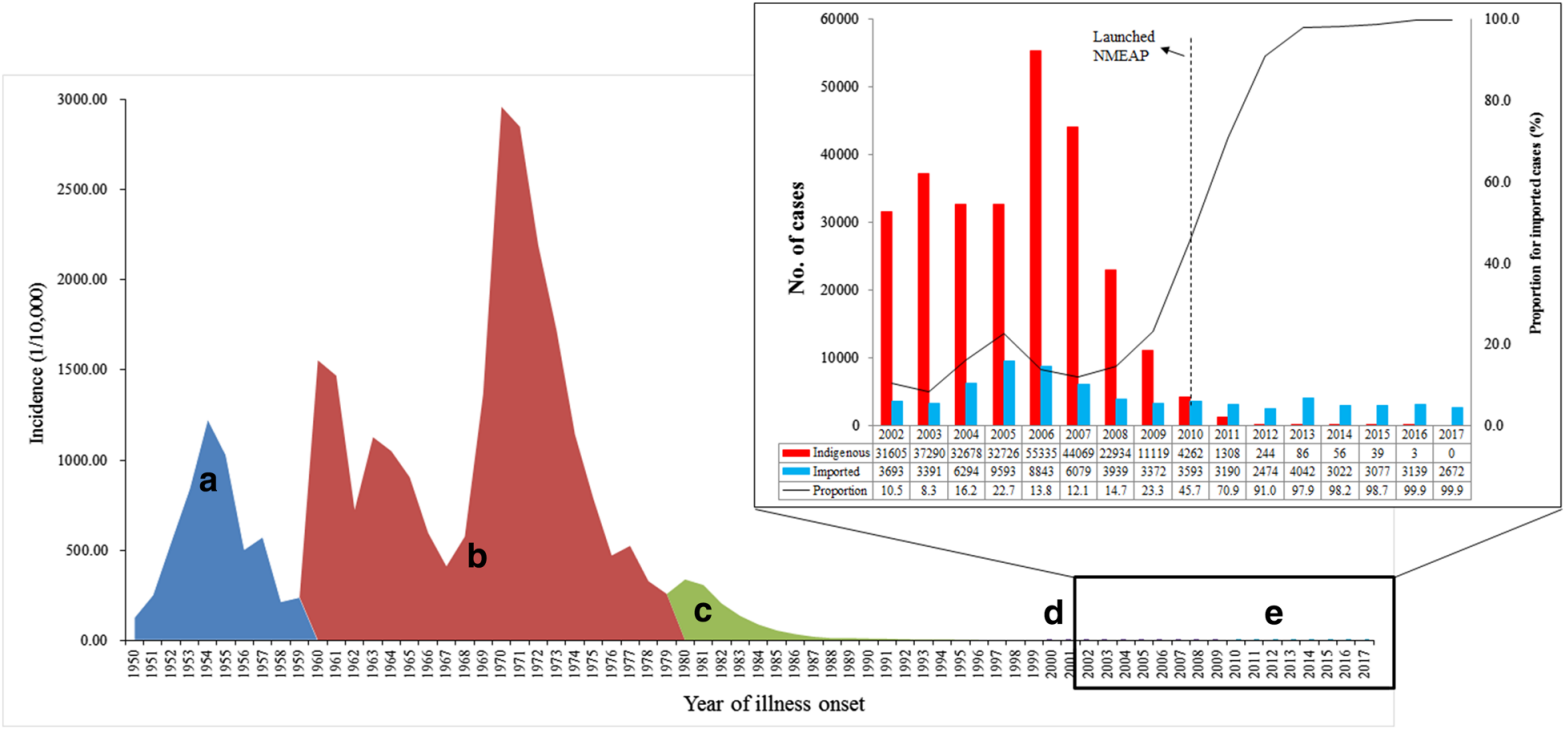
Incidence of malaria in China, 1950–2017. The different control and elimination phases are shown in different colours. **a** Transmission not known (1949–1959); **b** outbreak and pandemic transmission (1960–1979); **c** decline with sporadic distribution (1980–1999); **d** low transmission with re-emergence in central China (2000–2009); and **e** the elimination phase (2010–2020). The indigenous and imported cases from 2002 to 2017 are shown in the right-hand column

In the past, China suffered seriously from malaria epidemics [2]. Malaria has been documented in traditional Chinese medicine books, and the presence of malaria goes back approximately 4000 years in China’s history. Malaria expanded broadly, especially in rural regions, and outbreaks occurred often over the last few decades [3]. From the foundation of the People’s Republic of China in 1949 to reaching the goal of malaria free in 2020, the transmission of the disease can be primarily grouped into five phases: (i) transmission not known (1949–1959); (ii) outbreak and pandemic transmission (1960–1979); (iii) decline with sporadic distribution (1980–1999); (iv) low transmission with re-emergence in central China (2000–2009) [4]; and, (v) the elimination phase (2010–2020). Prior to 1949, there were approximately 30 million cases of malaria reported in China annually, and the mortality rate was about 1% [5]. Nevertheless, after the implementation of an integrated strategy for malaria control, including interventions, as well as socio-economic and environmental development, such as urbanization, alterations in the natural surroundings which affected the transmission pattern including changes of malaria vector distribution, the occurrence of indigenous malaria cases has been steeply reduced, and epidemic regions have drastically shrunk (Fig. 1) [6]. Thus, the former Ministry of Health, along with 13 additional ministries, issued the National Malaria Elimination Action Plan (2010–2020) (NMEAP), with the objective of eliminating indigenous malaria in non-border regions before the end of 2015 and eliminating the disease nationwide before the end of 2020 [7].

**Fig. 2:**
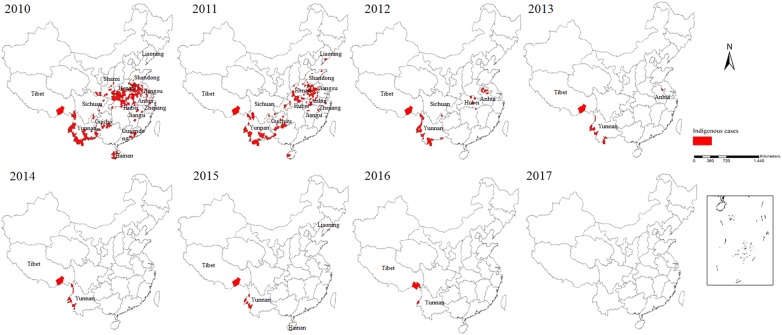
Indigenous malaria cases in China, 2010–2017. The red zones represent the areas where indigenous cases occur (the county level)

There is strong evidence that the country has reached crucial milestones towards malaria elimination over the last 8 years [8]. Firstly, from 2010, the transmission of malaria, which was primarily reported in 75 counties from 7 provinces, including Hainan and Yunnan, declined quickly in the first 4 years (Fig. 2) [9], while since 2014, malaria foci were mainly concentrated on the counties along the China–Myanmar border and in the Motuo County of the Tibetan Autonomous Region [10]. Secondly, a total of 5999 indigenous malaria cases were documented from 2010 to 2016, however those indigenous cases were steeply reduced by 99.9% over the 6 years (2010, n = 4262; 2016, n = 3) [11–17]. Thirdly, the species of malaria parasites that were transmitted has altered, e.g. no indigenous *Plasmodium malariae* and *Plasmodium ovale* were documented over the last few years, and the final indigenous case of *Plasmodium falciparum* was recorded in Canyuan County, Yunnan Province, in 2015. Fourthly, there were indigenous cases reported in 303 counties from 18 provinces in 2010, but there was zero indigenous case reported nationwide in 2017 [18] (Fig. 2). Fifthly, malaria elimination efforts have paid attention on the border areas since 2016, which was documented in the National Malaria Elimination Work Plan (2016–2020) issued by National Health Commission (NHC) of China with the interventions focusing on the clearance of active foci in Tibet and Yunnan provinces [19]. Finally, some innovative interventions were implemented in the border areas, such as establishment of ‘three border defensive lines’ to prevent re-establishment of malaria transmission due to imported cases in Yunnan border areas, and the malaria elimination programme and reference laboratory were established in remote areas of Tibet in 2016 to focus on case detection, management, foci investigation and response, in collaboration with professionals from the Guangdong Provincial Centre for Disease Control and Prevention.

In May 2015, the World Health Assembly endorsed a new Global Technical Strategy for Malaria 2016–2030 (GTS) [20]. One of the key target of the GTS is the elimination of malaria in at least 35 countries by 2030, and to keep that timeline in sight, the strategy established milestones along the way, including eliminating malaria by 2020 in at least 10 countries that had the disease in 2015. The GTS recognizes progression towards malaria-free status is a continuous process, and not a set of independent stages. Therefore, this review summarizes lessons learnt from the Chinese national malaria elimination programme and proposes further efforts to mitigate the challenges that still exist during the elimination phase, based on the analysis of malaria incidence patterns since 1950 and changes of the case-based malaria reporting data since 2010. The outcomes are likely to demonstrate that malaria elimination is a country-led and country-owned endeavour which can probably help improve malaria surveillance systems not only in China, but also in other elimination countries.

## Methods

### Data collection

A retrospective analysis was performed to assess the changes in malaria epidemic patterns from 1 January, 1950 to 31 December, 2017 at national level. The data before 2005 were obtained from annually paper-based surveillance data. The paper-based data were collected from each province, sent by mail to National Institute of Parasitic Diseases (NIPD) on early February in the next year. Since 2005, each of the separate cases from the Infectious Diseases Information Reporting Management System (IDIRMS, http://chinacdc.cn) was closely examined and scrutinized. IDIRMS is a standardized platform that assists health care systems across China in detecting, evaluating, preventing, and reacting to any communicable disease. The IDIRMS mandated that each of the confirmed and suspected malaria cases in China’s hospital system be documented with the local Centre for Disease Control and Prevention (CDC), and in turn, CDC staff to rigorously follow-up on these cases. An additional documenting system, the National Information Management System for Malaria (NIMSM, http://chinacdc.cn), established in 2012 to document the interventions, was also examined to complete data from IDIRMS. The ‘1-3-7’ approach, which refers to the case reporting within 24 h, case verification and investigation within 3 days, and foci investigation and response to inhibit the secondary transmission within 7 days, was established to deliver and monitor the elimination process across the whole country [21]. For ‘1’, this mandated the local health staff to report the confirmed and suspected cases within 24 h via a web-based reporting system. Then, a short message service system alerts local CDC staff via cell phone to perform the case follow-up in an expedited manner. There are two parts for ‘3’: one is case verification by microscopy and polymerase chain reaction (PCR) at high level such as provincial reference laboratory, and the other is case classification, which requires the health staff to determine whether the case was indigenous or imported. There are two parts for ‘7’: the first step is to investigate the focus and classification, the second step is to implement the response activities based on the results of the investigation. Reactive case detection, targeted anti-malarial administration, indoor residual spray, and information, education, and communication activities can be chosen in the response activity, non-residual or cleared up focus [22].

Once the cases was reported in IDIRMS, the information was also auto-register to the NIMSM after 5 min. Both the IDIRMS and the NIMSM are private. Malaria Department in NIPD was responsible for handling the national malaria data for these two systems, and has permission to access them. The data could be obtained once the staff entered a user name and password, and choose the selection variables.

### Data analysis

The data for the evaluation were chosen by utilizing the variables, including reviewing data and reporting area, but the data from Hong Kong, Macao and Taiwan were not included in these statistics. First, the variables, including geographical location, *Plasmodium* species, gender, and age of the cases were extracted from the IDIRMS database. Each case was examined by staff members from a local responsible institution, such as county CDC, to determine if the case was indigenous or imported. Second, the variables, such as date of diagnosis, date of reporting, date of the case evaluation, case classification (indigenous, imported, induced or introduced), focus of the investigation, and foci responses, were retrieved from the NMISM database. Finally, each of the documented malaria cases was geo-coded and paired to the county-level layers of polygon and point with ArcGIS 10.1 software (Environmental Systems Research Institute, Inc, Redlands, CA, USA).

## Results

### Trends of malaria incidence in China

China is the most populous country in the world with a population of around 1.4 billion. Mainland China is administratively divided into 31 areas that include provinces, municipalities and autonomous regions, which can be sub-divided into 2858 counties. Malaria was once a great challenge for the public health system and after sustainable efforts, the occurrence of indigenous malaria has been steeply reduced, and malaria-epidemic regions have been substantially shrunk between 1950 and 2017. There were approximately 30 million malaria cases yearly nationwide prior to 1949, and the incidence peak occurred in 1970 (2961/100,000). *Plasmodium vivax* was the major species for relatively long time. From 2004 to 2017, it accounted for 70.1% of all reported malaria cases and had a peak in 2006, particularly in Huang-Huai Plain of central China such as Anhui and Henan provinces, where the number of malaria cases in those two provinces accounted for 62.5% of the total cases in the country for that year [10–16, 23–29]. Since 2008, the number of *P. vivax* cases decreased while the proportion of *P. falciparum* increased sharply. For example, there are only 1033 *P. falciparum* cases reported in 2009, accounting for 7.1% of all reported cases, while in 2016, *P. falciparum* cases were 2066, and accounted for 65.7% of all reported cases [16].

For local transmission, through great efforts in different control phases, the disease burden sharply reduced and only 11,119 indigenous cases were reported in 2009. Substantial efforts were invested in different control intervention measures, and the disease burden was sharply reduced by 99.9% compared to 2010 when the national malaria elimination programme initiated. Indigenous cases were mainly documented in 2 regions since 2014, and in 2 counties from 2 provinces in 2016 [8, 30]. From 2005 to 2016, most indigenous cases (92.5%) were reported in Anhui (n = 2326 [38.8%]), Yunnan (n = 1375 [22.9%]), Henan (n = 930 [15.5%]), Hubei (n = 459 [7.7%]), and Guizhou (n = 458 [7.6%]).

### Patterns of imported case

Out of 2675 malaria cases reported in 2017, 99.9% of them were imported cases with only 0.1% (n = 3) induced cases from blood transfusion reported in Jiangsu (n = 2) and in Guangdong provinces (n = 1).

**Fig. 3:**
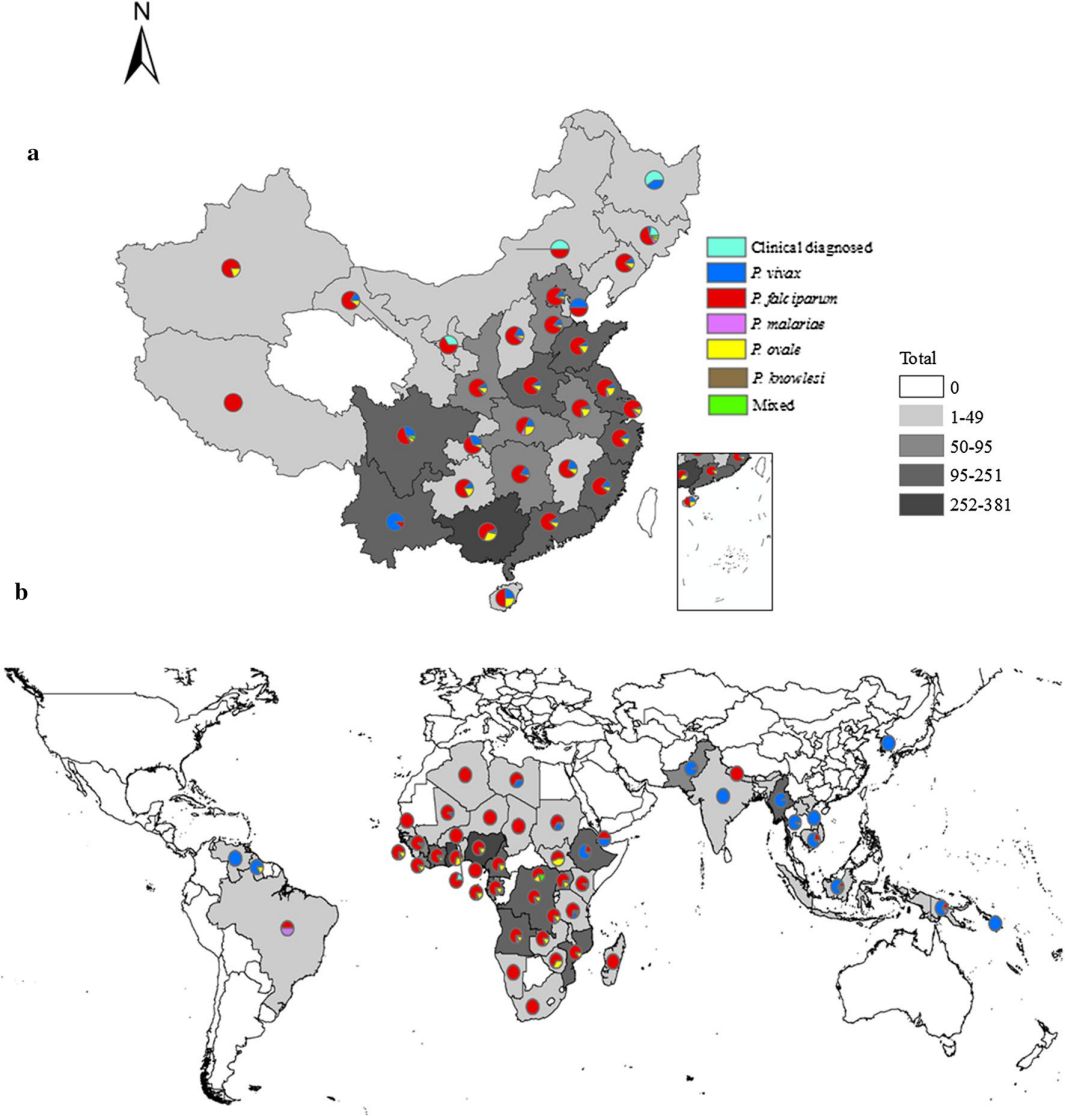
The distribution of imported cases in China stratified by species (**a**) and origin of those imported cases (**b**) in 2017. The number of malaria cases using different colours to represent the imported cases distribution in China, as well as the origin of those cases. Each province in China and the origin country was marked with different colours on the map

A total of 5 *Plasmodium* species were identified in the imported cases, including *P. falciparum* (n = 1719 [64.3%]), *P. vivax* (n = 496 [18.6%]), *P. ovale* (n = 350 [13.1%]), *P. malariae* (n = 65 [2.4%]), and *P. knowlesi* (n = 1), as well as mixed infections (n = 35 [1.3%]). The mixed infections were diagnosed as *P. falciparum* plus *P. vivax* (n = 14), *P. falciparum* plus *P. ovale* (n = 10), *P. vivax* plus *P. ovale* (n = 9), *P. falciparum* plus *P. malariae* (n = 1), *P. ovale* plus *P. malariae* (n = 1). Among the imported cases, 9 (0.3%) were clinically diagnosed in Jilin (n = 4), Heilongjiang (n = 3), Inner Mongolia (n = 1), and Ningxia (n = 1) (Fig. 3a).

Those malaria cases were imported from 53 countries, mainly from African countries (38 countries, n = 2285 [85.5%]), particularly from western (n = 952 [35.6%]) and central Africa (n = 881 [33.0%]) (Additional file 1: Table S1). For *Plasmodium* species, *P. falciparum* was mainly imported from western (n = 741 [43.1%]) and central Africa (n = 663 [38.6%]), *P. vivax* was mainly from Southeast Asia (n = 264 [53.2%]), *P. malariae* and *P. ovale* were mainly from western (n = 178 [42.9%]) and central Africa (n = 172 [41.4%]), and one case of *P. knowlesi* was imported from Indonesia (Fig. 3b). Only 7 of these imported cases were in individuals who died of *P. falciparum* infection.

### Reference laboratory network system

The 24 provincial reference laboratories, established between 2011 and 2014, have achieved all of the requirements to be part of the network, including laboratory room, devices, organization, and technical demands. Qualitative assessment was conducted by 20 blood slides from external quality assessment programme for communicable diseases (malaria microscopy) in the WHO Western Pacific Region. PCR assessment was carried out using 3 different blood spots to each staff in a blind test for analysis [31].

In addition, WHO has held external competency assessment of malaria microscopists in China since 2015, to assess microscopists, especially those at provincial level from endemic provinces. Currently, with increasing imported cases that are also distributed in non-endemic provinces, more and more staff from non-endemic provinces are required to participate in the WHO external assessment. Moreover, 2 rounds of the external quality assessment programme for malaria microscopy in NIPD were also organized annually sponsored by the WHO in 2013–2015. To date, 19 CDC staff obtained 1st level certificate and another 13 CDC staff obtained 2nd level certificate.

### Regional and inter-sectoral collaboration

The Middle Five Provinces Malaria Joint Control and Prevention Programme (Jiangsu, Anhui, Shandong, Hubei, Henan) was launched in 1974 and the Southern Three Provinces Malaria Joint Control and Prevention (Guangdong, Guangxi, Hainan) was launched in 1992 [32–34]; these are typical models for regional cooperation to greatly reduce malaria incidence in these regions. The regional collaboration mechanism strengthens timely information sharing and exchange on the mobile population and improves the capacity of medical staff for case management and foci response, which in turn facilitates the elimination process.

### Verification and assessment for malaria elimination

**Fig. 4:**
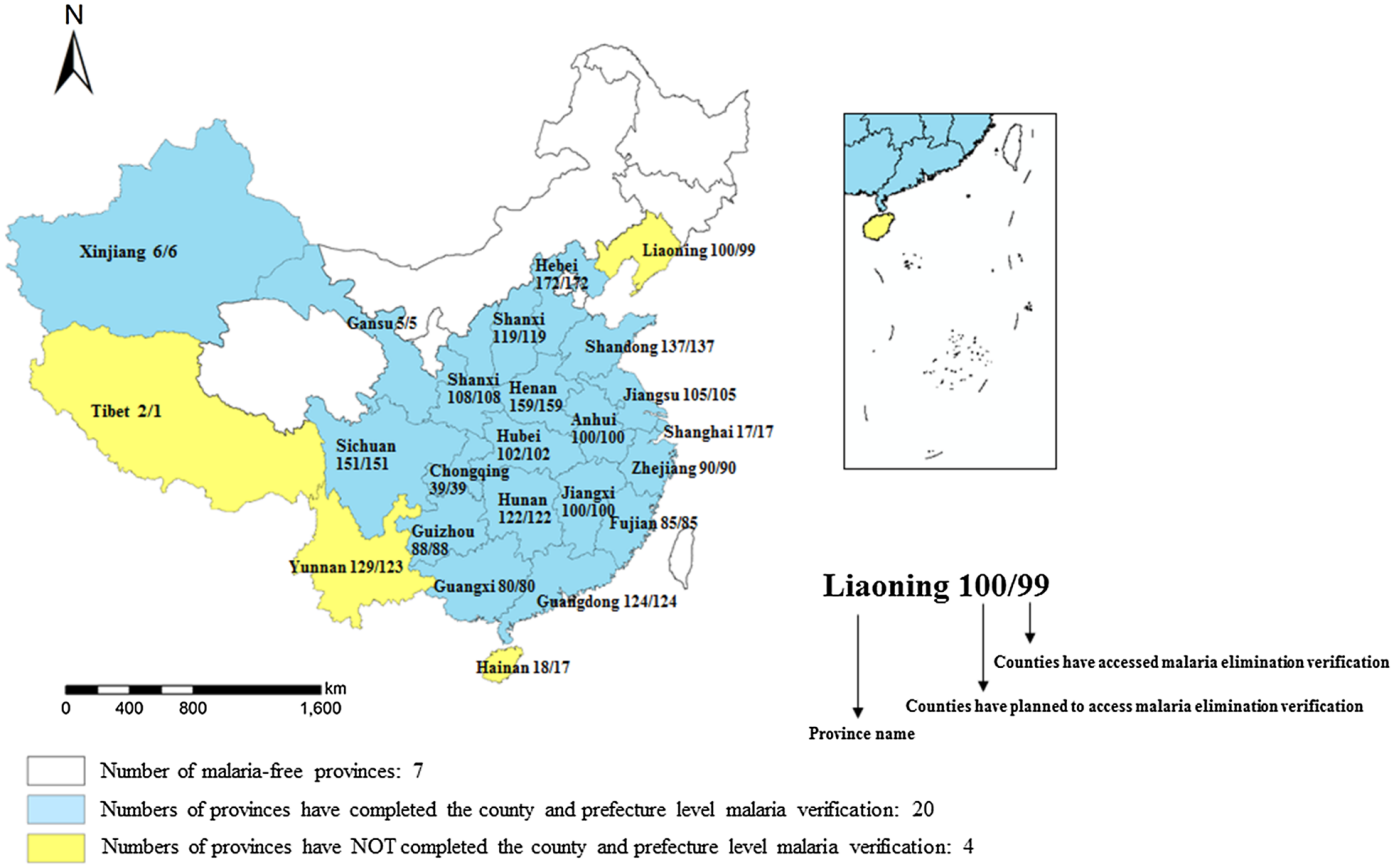
Sub-national malaria verification progress in China, 2012–2017. China has conducted sub-national verification of malaria elimination for all endemic provinces, and endemic provinces carry out county and prefecture malaria elimination verification by themselves. Similarly, a province/prefecture/county could conduct malaria elimination where no indigenous cases were reported in 3 consecutive years

Up to the end of 2017, a total of 2155 endemic counties and 215 prefectures completed the malaria verification, which accounts for 99.5% and 83.3% of all of the endemic counties and prefectures, respectively (Fig. 4). In 2015 and 2018, Shanghai and Jiangxi achieved sub-national malaria verification by the NHC [35].

### Monitoring and evaluation

In 2017, the NHC established two national expert groups: the National Experts Group on Malaria Elimination, and National Experts Group on Severe Malaria. Every quarter, the national elimination expert group carefully reviewed reported malaria cases, mainly on case investigation and foci response. The national experts group on severe malaria reviewed each death case attributed to malaria since 2016 and analysed the cause and lessons learned from those reports.

Each year, the NHC organizes panels of experts on malaria elimination to supervise the elimination process for endemic provinces. The provinces are selected because they still harbour active foci, exhibit increasing imported cases reported, or the interventions were not well implemented. The experts review the data quality, especially on case diagnosis, investigation, and foci response, and carry out field visits to the county. The experts provide technical support and feedback to the local government, bureau of health, CDCs, and provide a summarized report to the NHC.

## Discussion

Achieving universal health coverage (UHC) is one of the targets the nations of the world set when adopting the Sustainable Development Goals in 2015. Countries that progress towards UHC will make progress towards other health-related targets, such as end of malaria epidemic by sustained surveillance and prompt response for each malaria foci. In spite of the fact that China has made large strides in managing malaria over the last several decades, zero case of indigenous malaria was reported in the whole county in 2017, which indicated that Chinese NMEAP has entered into a new era to intensively sustain zero local transmission of the disease [18]. Therefore, it is the right time to answer the following two questions: What are the major lessons from Chinese NMEAP? What are the challenges of sustaining malaria non-transmission status?

### Lessons learnt from the successive NMEAP

The success of NMEAP since 2010 is mainly due to two different strategies. One is the country leadership with clear national elimination strategy, supported by two systems, namely case-based surveillance and response system and reference laboratory system. The other is the country-led efforts with regional and inter-sectoral collaboration as well as sustained monitoring and evaluation.

### Country leadership efforts

#### National elimination strategy

**Fig. 5:**
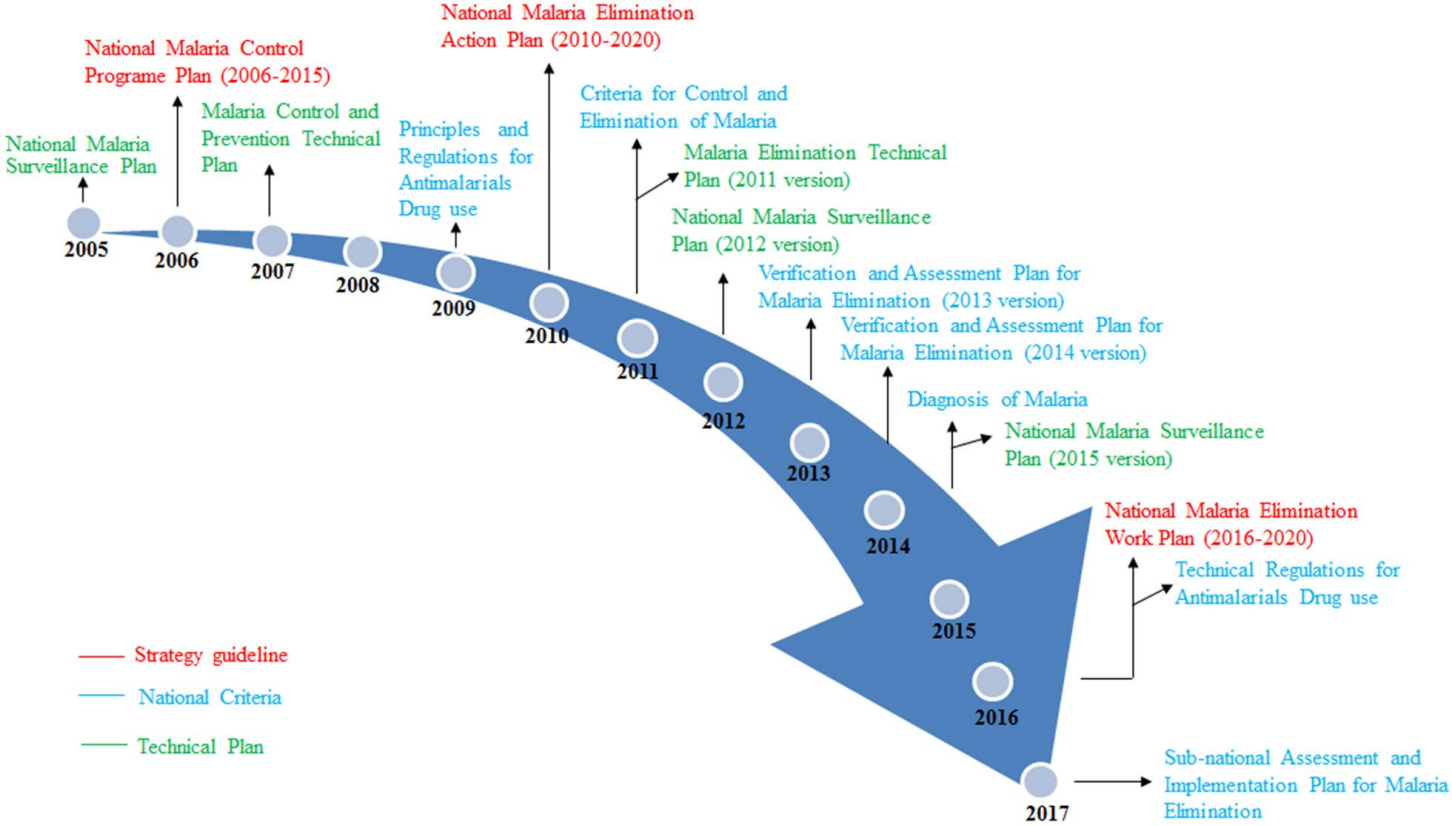
Documents developed to guide the national malaria control and elimination programme, 2005–2017

Strong governmental leadership is essential for a malaria control and elimination programme. Between 2005 and 2017, the central government developed a series of documents, including 3 strategy guidelines, 7 national criteria, and 5 technical plans (Fig. 5). These documents helped staff members to make appropriate decisions when malaria was at epidemic proportions or even if there was an outbreak during control and elimination phase. For example, since malaria incidence has been at the lowest level in 2009, the former Ministry of Health developed a new stratification of malaria endemicity at the county level, which stated in NMEAP that each of the counties in China was divided into 4 types, with its own strategy and interventions.

The central government unified command scheduling, to mobilize social resources to participate in the prevention and treatment of malaria. Local government officials support and prioritize malaria control and elimination when they are given specific malaria control and elimination targets. The local Bureau of Health has developed its own malaria elimination goal and timetable.

#### Community mobilization

The government mobilized social and financial resources for malaria control and elimination. The mobilization of various sectors, including the community and the technical staff in institutions and control services to participate and cooperate, is one of the major experience for the malaria control programme [36]. For instance, China has experience with mass drug administration (MDA) of pyrimethamine and primaquine. In the period from 1970s to 1980s, MDA was widely performed on a large scale involving millions of people in Southern China, to decrease malaria incidence of *P. vivax* [37]. After 2000, focal MDA was adopted on a smaller scale, usually implemented in the foci response focus on households and natural villages.

### Case-based surveillance and response system (individual case tracking and focus clearing)

China has experienced a big transition with its surveillance system. Between 1950 and 1985, malaria cases were recorded via county-based monthly reports delivered by post; in 1985–2003, malaria cases were recorded monthly via electronic means, and in 2004, due to the severe acute respiratory syndrome outbreak, the real-time information reporting management system, IDIRMS, was established [38].

After 2010, the national malaria surveillance and response system has been altered so it is in line with the transition from control to elimination phase to promptly detect and investigate each of the malaria infections and be sure that the appropriate treatment is administered, in order to prevent re-establishment. An additional reporting system, NMISM, was established in 2012 to observe and document cases within the time frame indicators utilizing the ‘1-3-7’ approach. To date, good performance has been seen nationwide on ‘1-3-7’ activities (Table 1). The Chinese 1-3-7 approach has already proven to be successful but still needs to be improved, especially for prioritizing surveillance and response activities based on different risk characteristics of malaria transmission [39]. Even so, the 1-3-7 approach could be considered a model for other countries.

**Table 1:** Implementation of ‘1-3-7’ elimination approach in China, 2013–2017

Year	Rate of cases report within 1 day (%)	Rate of laboratory detection^a^ (%)	Rate of laboratory confirmation^b^ (%)	Rate of epidemiological investigation within 3 days (%)	Number of foci disposed within 7 days (%)
2013	4128 (100.0)	4028 (97.6)	3949 (95.7)	3966 (96.1)	1687 (40.9)
2014	3078 (100.0)	3068 (99.7)	3021 (98.2)	3046 (99.0)	2118 (68.8)
2015	3116 (100.0)	3093 (99.3)	3058 (98.1)	3085 (99.0)	2215 (71.1)
2016	3143 (100.0)	3134 (99.7)	3129 (99.6)	3096 (98.5)	2290 (72.9)
2017	2675 (100.0)	2675 (100.0)	2666 (99.7)	1685 (83.6)	1949 (72.9)

^a^The number (proportion) of cases detected in the laboratory using microscopy, RDTs or PCR

^b^The number (proportion) of cases confirmed as malaria by the method of microscopy or PCR in provincial reference laboratory

### Reference laboratory system

A malaria diagnostic reference laboratory system covering 24 endemic provinces has been set up in China. Its primary duties are case diagnosis and verification, capacity training, establishing a quality assurance sample bank, method innovation, and technical support at various levels to provide precise and reliable results, including species determination between *P. vivax* and *P. ovale*, which are easily confused when using only microscopy [40]. As of now, the determination of *Plasmodium* species via microscopy and PCR is at medium to high level [31]. The national malaria reference laboratory in NIPD, is responsible for investigating all difficult samples from provincial laboratory, as well as carrying out quality control for capacity building in 24 provincial reference laboratory.

The National Parasitic Technology March, which has been organized by the NHC since 2011, assesses parasitic knowledge, blood film preparation, and microscopy of medical professionals and CDC staff from all 31 provinces. In reference to malaria, from 2011 to 2015, more than 600 CDC staff participated in the microscopy examination and malaria control and prevention knowledge; this should improve the awareness of malaria management and prevention and increase clinicians’ skill in diagnosing and treating the disease.

### Country-led efforts

#### Regional and inter-sectoral collaboration

China has successfully carried out regional cooperation and collaboration for malaria control and elimination for several decades, such as the Middle Five Provinces Malaria Joint Control and Prevention Programme and the Southern Three Provinces Malaria Joint Control and Prevention Programme.

To improve surveillance and management of the increasing number of imported cases, inter-sectoral cooperation mechanism is needed which requires intense strengthening, especially for the entry-exit inspection and quarantine, departments of education, agriculture, immigration, and tourism. For example, the entry-exit inspection and quarantine departments have screened each fever patient using rapid diagnostic tests (RDTs) in airports or customs posts. Once they have obtained a positive result, staff will send a patient to the nearest CDC or designated hospital for further treatment. Meanwhile, staff will conduct an epidemiological investigation, collect the blood sample and send it to the laboratory for confirmation, and report the results through the web-based system.

#### Sustained monitoring and evaluation

China’s CDC has further standardized data management, including data collection, verification, and analysis. Daily reports, including analysis and real-time mapping of case distribution on the internet, monthly data collection of sentinel surveillance, quarterly video meeting for elimination progress, and, annual dissemination meeting and publications, will all be summarized and fedback to the NHC and all CDC levels.

#### Sustained financial support

The Global Fund to Fight AIDS, Tuberculosis and Malaria (GFATM) was one of the largest funder for malaria control and elimination in the last decade in China. From 2002 to 2012, China has successfully obtained GFATM support for rounds 1, 5, 6, and 10. The Global Fund-supported projects were implemented in 20 provinces in 2010, and a total of US$116 million were released, which was used to assist China in reaching its achievement of malaria elimination goal. With the GFATM support, almost 1.4 million malaria cases were treated, 2.80 million insecticide-treated bed nets (ITNs), and 1.80 million long-lasting insecticide nets (LLINs) were distributed [41].

As the national strategy implementation was completed before the 30 June, 2012 deadline, the Chinese government provided the missing funds. Between 2013 and 2015, a total of US$51.6 million were predominantly allocated for diagnostic testing (58%) and management and other costs (28%). The expenses of ITNs and LLINs, insecticides, spraying solutions, and anti-malarial medicines took up limited proportions of the international (4%) and domestic (13%) funding [42].

### Challenges for malaria resurgence

Efforts in pursuing a malaria-free world have never waned. In spite of the stalled progress globally, there is an increasing number of countries that are close to achieving malaria elimination: while there were 37 countries with fewer than 10,000 indigenous malaria cases in 2010 this number had increased to 44 by 2017 [1]. In China, although great successes in malaria management and elimination have been attained, there are still challenges in the elimination process or post-elimination process, including how to maintain the non-transmission status, how to implement the qualified verification and assessment, how to overcome the difficulties in border areas through regional cooperation.

### Maintenance of non-transmission status

A country can be assessed for WHO certification of malaria elimination following the documentation of zero indigenous case of malaria for a minimum of 3 consecutive years. To keep non-malaria status from 2018–2020, China still has a long way to go.

Once the sustained importance on elimination interventions has waned, malaria could be re-introduced in areas where it has been previously eliminated, because the *Anopheles* mosquito still exists. For instance, in Liaoning and Hainan, after malaria transmission was interrupted for 4 years, officials reported 2 local *P. vivax* and 6 local *P. malariae* cases, respectively, in 2015 [6].

Malaria elimination is a big challenge at the border between Yunnan and Tibet provinces, as there are many vectors that can transmit malaria in the area. There is a big mobile cross-border population and there is no natural barrier in the area, which makes surveillance of all imported malaria cases a huge challenge [43–45]. Further, inadequate transportation also makes it difficult to perform epidemiological investigation and blood smear confirmation within 3 days. To solve this problem, the ‘three border defensive lines’ programme was implemented and aims at preventing malaria re-establishment, by establishing 68 malaria and consultation service posts in the second defence line, by providing RDTs to screen for patients with fever, and planning the development of a new case detection technology for migrant population [46]. Besides, Yunnan Institute of Parasitic Diseases has established a work station in Nabang Village (a village of Yingjiang County, neighbouring Myanmar) to carry out diagnosis using a high-throughput screening method based on 18 sRNA throughout the transmission season [47].

Given the reason that the possibility of resurgence of malaria still remain particularly for *P. vivax*, due to the widespread distribution of *Anopheles sinensis* in China. Therefore, epidemiological and entomological surveillance, early and quickly response to the imported cases and prevention from onward transmission will certainly required. It is still necessary for China to keep vigilance on the malaria resurgence to guide health policy in monitoring and preventing potential risks of malaria resurgence or outbreaks.

### Surveillance and response to the imported malaria

Imported cases are another big challenge to face in the elimination phase. In comparison to the steep reduction of indigenous cases, the imported malaria cases constituted 16.2% of the total cases reported in 2004, but in 2017, this had increased to 99.9%. In 2013, when clustered migrant employees returned from Ghana to Shanglin County of the Guangxi Zhuang Autonomous Region, about 1000 imported malaria cases found from migrants, significantly lifting the nationwide reported malaria numbers that year [48, 49]. To deal with this situation, a risk assessment should be well conducted, particularly in areas that harbour the *An. sinensis*. Information sharing among sectors and departments should be established. In the areas where imported cases are clustered, CDC staff can carry out health education programmes in the villages and townships where returning workers reside, at airports, and at hospitals, through TV, radio, mobile phone messages, and the delivery of educational information materials. Furthermore, there is an urgent need to improve the effectiveness of the different interventions to decrease the costs of imported cases management [50].

Surveillance and response system should be maintained to be effective in the elimination and post-elimination stages, focusing on hotspots and risk populations, to avoid any transmission and prevent re-establishment. China’s 1-3-7 strategy is one of the methods to practice reactive surveillance and reaction, and it has been proven to be efficient in lowering transmission, even on the China–Myanmar border of Yunnan Province [51]. Great attention should be paid to non-residual foci, which refer to transmissions interrupted recently (within the last 1–3 years). Capacity building should be continuously maintained, especially for the timely detection, appropriate diagnosis and treatment, as well as foci investigation and response.

### Verification and assessment for malaria elimination

China was listed as one of the countries at an elimination stage in the WHO World Malaria Report 2015 [52]. To fulfill and assess the elimination process and achieve the goal of certification by WHO, China has adopted county, prefecture and sub-national verification since 2012. From 2018, the NHC will organize national expert panels from related departments to conduct sub-national malaria verification on the endemic provinces.

### Global cooperation and engagement

China is on track to eliminate malaria and the experience gained could be used by other countries that are on the same path. There is a need for collaboration to control and eliminate malaria in those countries which have shared imported malaria cases with China [53].

To reduce the malaria burden on the China–Myanmar border, since 2010, county CDC staff on China side and Myanmar have carried out joint control and prevention work mechanism, particularly on information sharing, with regard to border-specific mobile population management and surveillance system, to lower the malaria incidence on the China–Myanmar border region. Under bilateral or multilateral cooperative mechanisms, collaboration in strengthening joint prevention and control interventions, carrying out regular information exchange by bilateral meetings, and establishing joint coordination and steering committee have been set forth [54]. This work eased information exchange and communication with 5 state special areas on the Myanmar side, including Kachin State Special Region, Shan State Special Region, 1st Wa Bang State Special Region, Guo Gan State Special Region, and 1st Kachin State Special Region. Health Poverty Action, an integral member of the China–Myanmar malaria management and elimination project, eased the formation of an information exchange platform for observing and reacting on both sides [55]. In addition, drug resistance research is also being conducted to contain the spread of artemisinin-based combination therapy-resistant isolates of *P. falciparum*, which emerged on the Thai-Cambodian border in the Great Mekong Sub-region, which may be catastrophic to malaria elimination in border countries [56–58]. Some work previously conducted and reported on drug resistance is still monitored and strengthened in the elimination phase [59, 60].

China now cooperates with countries in Africa and Southeast Asia under the framework of the ‘Belt and Road’ initiative [61]. For example, the China-UK-Tanzania malaria control application of community-based and integrated strategy, which is a pilot project to improve malaria diagnosis, reporting, treatment, and tracking using China’s 1-3-7 model and surveillance systems, will explore the model for effectively reducing local malaria disease burden by applying Chinese knowledge and experience with malaria control integrated with the WHO-T3 strategy (Test, Treat and Track) in the pilot areas [62].

## Conclusion

No indigenous case was reported in China in 2017, and most of the malaria cases (2672 cases) are imported from African and Southeast Asian countries at the same time [18]. Strategies and interventions have been developed and implemented to facilitate the national malaria elimination programme in order to achieve the goal of malaria elimination, with the evidence that no indigenous case being reported for at least 3 consecutive years in the country. Nevertheless, to deal with the elevated number of imported cases and retain the free-malaria status in whole country, it is necessary to intensify multi-interventions, including governmental leadership, a pro-active surveillance system, capacity building, maintain fund support, multi-sectoral cooperation, and community mobilization, to make sure that every case is quickly detected and appropriate treatment is administered once the case is confirmed, during the elimination and post-elimination phases of the national programme. China’s engagement and leadership in regional malaria elimination initiatives has helped to promote the progress of malaria elimination efforts in neighbouring countries where there is malaria transmission. China is beginning to cooperate with Africa countries on malaria control and elimination in order to reduce the number of imported malaria cases further. Building on its 2017 milestone of zero indigenous cases, and backed by strong national commitment, China is on a solid footing towards the goal of malaria elimination by 2020.

## Additional file

**Additional file 1: Table S1.** Origin of the countries for the imported cases in China, 2017.
