# Supplementary material for: Ready for malaria elimination: zero indigenous case reported in the People’s Republic of China
Source: Malar J. 2018 Aug 29;17:315. doi: 10.1186/s12936-018-2444-9 (PMC6116478; doi:10.1186/s12936-018-2444-9)
Supplement: Supplementary file 1 — Additional file 1: Table S1. Origin of the countries for the imported cases in China, 2017. [file 12936_2018_2444_MOESM1_ESM.doc]

**Table S1 Origin of the countries for the imported cases in China, 2017.**

| **Country of origin** | **Total** | **Clinical** | ***P. falciparum*** | ***P. vivax*** | ***P. malariae*** | ***P. ovale*** | ***P. knowledsi*** | **Mixed infections** |
| --- | --- | --- | --- | --- | --- | --- | --- | --- |
| **Africa** | **2285** | **9** | **1692** | **147** | **60** | **345** | **0** | **32** |
| Ghana | 347 | 1 | 243 | 5 | 3 | 93 | 0 | 2 |
| Angola | 195 | 1 | 146 | 3 | 15 | 27 | 0 | 3 |
| Equatorial Guinea | 132 | 0 | 100 | 5 | 3 | 23 | 0 | 1 |
| Nigeria | 286 | 0 | 225 | 8 | 6 | 46 | 0 | 1 |
| Congo (Brazzaville) | 119 | 1 | 82 | 4 | 3 | 26 | 0 | 3 |
| Guinea | 80 | 0 | 72 | 3 | 2 | 2 | 0 | 1 |
| Cameroon | 190 | 2 | 137 | 7 | 6 | 34 | 0 | 4 |
| Liberia | 61 | 0 | 50 | 2 | 0 | 8 | 0 | 1 |
| Sudan | 15 | 0 | 10 | 4 | 1 | 0 | 0 | 0 |
| Ethiopia | 113 | 1 | 24 | 78 | 1 | 4 | 0 | 5 |
| Sierra Leone | 44 | 0 | 34 | 1 | 3 | 6 | 0 | 0 |
| Gabon | 53 | 1 | 40 | 2 | 3 | 7 | 0 | 0 |
| Congo (Kinshasa) | 151 | 0 | 118 | 8 | 5 | 19 | 0 | 1 |
| Mozambique | 103 | 1 | 87 | 0 | 1 | 14 | 0 | 0 |
| Zambia | 30 | 0 | 24 | 1 | 0 | 5 | 0 | 0 |
| Tanzania | 46 | 0 | 34 | 7 | 3 | 2 | 0 | 0 |
| Uganda | 66 | 0 | 48 | 1 | 3 | 11 | 0 | 3 |
| Chad | 13 | 0 | 13 | 0 | 0 | 0 | 0 | 0 |
| South Sudan | 9 | 0 | 5 | 0 | 0 | 4 | 0 | 0 |
| Côte d’Ivoire | 97 | 0 | 82 | 1 | 1 | 8 | 0 | 5 |
| South Africa | 8 | 0 | 8 | 0 | 0 | 0 | 0 | 0 |
| Mali | 13 | 0 | 12 | 1 | 0 | 0 | 0 | 0 |
| Togo | 5 | 1 | 4 | 0 | 0 | 0 | 0 | 0 |
| Malawi | 13 | 0 | 10 | 0 | 1 | 2 | 0 | 0 |
| Benin | 9 | 0 | 9 | 0 | 0 | 0 | 0 | 0 |
| Libya | 3 | 0 | 2 | 1 | 0 | 0 | 0 | 0 |
| Madagascar | 8 | 0 | 8 | 0 | 0 | 0 | 0 | 0 |
| Kenya | 23 | 0 | 21 | 0 | 0 | 1 | 0 | 1 |
| Niger | 4 | 0 | 4 | 0 | 0 | 0 | 0 | 0 |
| The Central African Republic | 28 | 0 | 27 | 0 | 0 | 1 | 0 | 0 |
| Burkina Faso | 4 | 0 | 4 | 0 | 0 | 0 | 0 | 0 |
| Senegal | 1 | 0 | 1 | 0 | 0 | 0 | 0 | 0 |
| Zimbabwe | 3 | 0 | 2 | 0 | 0 | 1 | 0 | 0 |
| Rwanda | 5 | 0 | 3 | 0 | 0 | 1 | 0 | 1 |
| Algeria | 1 | 0 | 1 | 0 | 0 | 0 | 0 | 0 |
| Namibia | 1 | 0 | 1 | 0 | 0 | 0 | 0 | 0 |
| Djibouti | 2 | 0 | 1 | 1 | 0 | 0 | 0 | 0 |
| Burundi | 4 | 0 | 0 | 4 | 0 | 0 | 0 | 0 |
| **Southeast Asia** | **291** | **0** | **19** | **264** | **1** | **3** | **1** | **3** |
| Myanmar | 245 | 0 | 14 | 228 | 1 | 0 | 0 | 2 |
| Cambodia | 14 | 0 | 3 | 10 | 0 | 1 | 0 | 0 |
| Indonesia | 18 | 0 | 2 | 13 | 0 | 1 | 1 | 1 |
| Laos | 13 | 0 | 0 | 12 | 0 | 1 | 0 | 0 |
| Vietnam | 1 | 0 | 0 | 1 | 0 | 0 | 0 | 0 |
| **Southern Asia** | **67** | **0** | **3** | **63** | **1** | **0** | **0** | **0** |
| Pakistan | 63 | 0 | 2 | 60 | 1 | 0 | 0 | 0 |
| India | 3 | 0 | 0 | 3 | 0 | 0 | 0 | 0 |
| Nepal | 1 | 0 | 1 | 0 | 0 | 0 | 0 | 0 |
| **Eastern Asia** | **1** | 0 | **0** | **1** | **0** | 0 | 0 | 0 |
| Korea | 1 | 0 | 0 | 1 | 0 | 0 | 0 | 0 |
| **Oceania** | **21** | **0** | **3** | **15** | **2** | **1** | **0** | **0** |
| Papua New Guinea | 19 | 0 | 3 | 13 | 2 | 1 | 0 | 0 |
| Solomon Islands | 2 | 0 | 0 | 2 | 0 | 0 | 0 | 0 |
| **Latin America** | **10** | **0** | **2** | **6** | **1** | **1** | **0** | **0** |
| Guyana | 6 | 0 | 0 | 5 | 0 | 1 | 0 | 0 |
| Ecuador | 1 | 0 | 1 | 0 | 0 | 0 | 0 | 0 |
| Venezuela | 1 | 0 | 0 | 1 | 0 | 0 | 0 | 0 |
| Brazil | 2 | 0 | 1 | 0 | 1 | 0 | 0 | 0 |
| **Total** | **2675** | **9** | **1719** | **496** | **65** | **350** | **1** | **35** |
